# Nutritional Characterization of Sea Bass Processing By-Products

**DOI:** 10.3390/biom10020232

**Published:** 2020-02-04

**Authors:** Paulo E. S. Munekata, Mirian Pateiro, Rubén Domínguez, Jianjun Zhou, Francisco J. Barba, Jose M. Lorenzo

**Affiliations:** 1 Centro Tecnolóxico da Carne de Galicia, rúa Galicia n° 4, Parque Tecnolóxico de Galicia, San Cibrao das Viñas, 32900 Ourense, Spain; paulosichetti@ceteca.net (P.E.S.M.); mirianpateiro@ceteca.net (M.P.); rubendominguez@ceteca.net (R.D.); 2 Nutrition and Food Science Area, Preventive Medicine and Public Health, Food Science, Toxicology and Forensic Medicine Department, Faculty of Pharmacy, Universitat de València, Avda. Vicent Andrés Estellés, s/n, 46100 Burjassot, València, Spain; jianz@alumni.uv.es (J.Z.); francisco.barba@uv.es (F.J.B.)

**Keywords:** *Dicentrarchus labrax*, by-product, amino acids, fatty acids, omega-3, minerals, calcium, phosphorus, biological relevance

## Abstract

The consumption of functional foods and nutraceuticals is gaining more importance in modern society. The exploration of alternative sources and the utilization of by-products coming from the food industry are gaining more importance. The present study aimed to characterize the nutritional value and potential use of sea bass by-products as a source of high-added-value compounds for the development of supplements. The chemical composition (moisture, protein, fat, and ash contents) and profiles of amino acids (high-performance liquid chromatography coupled to a scanning fluorescence detector), fatty acids (gas chromatography coupled to a flame ionization detector), and minerals (inductively coupled plasma optical emission spectroscopy) were determined for sea bass fillet and its by-products (skin, guts, gills, liver, head, and fish bones). The chemical composition assays revealed that by-products were rich sources of proteins (skin; 25.27 g/100 g), fat (guts and liver; 53.12 and 37.25 g/100 g, respectively), and minerals (gills, head, and fish bones; 5.81, 10.11, and 7.51 g/100 g, respectively). Regarding the amino-acid profile, the skin and liver were the main sources of essential amino acids with an essential amino-acid index of 208.22 and 208.07, respectively. In the case of the fatty-acid profile, all by-products displayed high amounts of unsaturated fatty acids, particularly monounsaturated (from 43.46 to 49.33 g/100 g fatty acids) and omega-3 fatty acids (in the range 10.85–14.10 g/100 g fatty acids). Finally, the evaluation of mineral profile indicated high contents of calcium and phosphorus in gills (1382.62 and 742.60 mg/100 g, respectively), head (2507.15 and 1277.01 mg/100 g, respectively), and fish bone (2093.26 and 1166.36 mg/100 g, respectively). Therefore, the main sources of monounsaturated, unsaturated, and long-chain omega-3 fatty acids were guts and liver. The most relevant source of minerals, particularly calcium, phosphorus, and manganese, were head, fish bones, and gills. The most promising source of proteins and amino acids was the skin of sea bass.

## 1. Introduction

The consumption of healthier and functional foods and nutraceuticals (foods or supplements that are associated with beneficial physiological effects) is an important part of modern society [1]. This important share of the food market is supported by consumers that are aware of the relationship between food composition and health. Essentially, the main motive among consumers to intake these products is the desire to reduce the risk associated with chronic diseases (main leading causes of death at a global scale) or to enhance health (improving the immune system or well-being) [2]. The biological effect obtained from functional foods and nutraceuticals is actually associated with a single or a group of compounds (present in their composition) that are naturally present or intentionally added to the product. Particularly for functional foods, these products can be classified as “fortified” (increased content of naturally occurring component) or “enriched” (adding an external component). In this sense, important dietary components were put in the spotlight, such as omega-3 (n-3) fatty acids, amino acids (such as arginine, leucine, and tyrosine), and minerals (calcium, phosphorus, and manganese, for instance) [3].

Aiming at the enrichment or fortification of food, functional ingredients can be obtained from natural sources, biotechnological generation, or chemical synthesis. For instance, omega-3 fatty acids can be found in fish oil (6–13 g C20:5n3 and 4–18 g C22:6n-3/100 g oil) and algae oil (10–95 g eicosapentaenoic acid—EPA/100 g oil and 20–45 g docosahexaenoic acid—DHA/100 g oil) [4,5]. At the commercial level, the most common n-3-rich products to produce functional food are fish and algae oil that can be found in liquid, microencapsulated, and emulsified forms [5,6]. In the case of amino acids, commercial production is carried out by fermentation of carbohydrates with specific bacterial strains such as *Corynebacterium glutamicum* and *Escherichia coli*, which can produce arginine and leucine [7]. Particularly for minerals (such as calcium, phosphorus, and manganese), the main current sources are organic and inorganic salts that are obtained from chemical synthesis [8]. Consequently, the application of functional ingredients in food was previously reported, for example, n-3 fatty acids were enriched in meat products [9], dairy products were fortified in calcium [10], and baked products were enriched in amino acids [11]. Furthermore, the development of dietary supplements such as n-3 fatty capsules is also widespread [12].

However, the increasing development of this sector created a demand for new/underutilized sources of these biologically relevant compounds [13]. Consequently, several researchers are re-evaluating the importance and potential use of food industry by-products [14,15], which can also reduce the environmental impact associated with this industrial sector. In this sense, the development and implementation of strategies to reuse food industry by-products is a necessary action to improve the efficiency of industrial operations, reduce the discard of wastes, and also recover high-added value compounds for further utilization in food products, which fulfill the concepts of sustainable circular economy [13]. Particularly for the fish industry, the by-products such as viscera, skin, fish bones, and head are the main by-products generated from industrialization processes [16]. Although these by-products are discarded as a low-value material, several studies reported the importance of exploring the by-products of fish processing as potential sources of functional ingredients. For instance, the fish skin is a rich source of amino acids [17], the fish liver is a relevant source of polyunsaturated fatty acids (PUFA), particularly long chain n-3 fatty acids [18], and the fish bone is rich in calcium [19].

This scenario supports the study of species largely consumed in key regions of the globe. With this line of thought, sea bass (*Dicentrarchus labrax*) can be seen as a relevant source of biologically relevant compounds. The sea bass is an edible fish largely found in the Mediterranean Sea and inshore waters of this region. This fish is also characterized by a silver-gray to a bluish color, small scales, and moderately forked caudal fin. The historical relevance of sea bass dates back to the 1960s when the culture of this fish was intensified, particularly in France and Italy. Moreover, the successful implementation of a culture system for sea bass larvae for mass products in the 1970s favored this species to became the first non-salmonid species to be widely produced in Europe and countries of the Mediterranean Sea [20]. The average per capita consumption of sea bass in the Mediterranean basin was around 200 g in 2016. Moreover, Greece, Portugal, and Cyprus were the leading countries in the consumption per capita of sea bass (796, 680, and 643 g), followed by Spain and Italy (545 and 513 g) [21].

Due to the scarce scientific information regarding the increasing demand for alternative, natural, and environmentally friendly sources of biologically relevant compounds, the present study aimed to characterize the chemical composition and profiles of amino acids, fatty acids, and minerals of sea bass by-products.

## 2. Materials and Methods

### 2.1. Samples

The present study was carried out with 10 sea bass fishes (full individuals) that were purchased in a local market in Ourense (Spain) and transported under refrigeration to the Centro Tecnológico de la Carne de Galicia (Ourense, Spain). The characteristics of animals are described as follows: average length of 35 cm (standard error of the mean (SEM) = 0.4); average weight of 512 g (SEM = 6); males around 36 months old; fishes were bought on five different occasions during October and November (2018). The fishes were filleted and dissected to obtain the following by-products: skin, guts, gills, liver, head, and fish bone. The fillets and each by-product were individually homogenized and kept at −80 °C until further analysis.

### 2.2. Proximate Composition

The evaluation of chemical composition was determined according to the International Organization for Standardization (ISO) recommended standards 1442:1997 [22] for moisture, 937:1978 [23] for protein content, and 936:1998 [24] for ash content. The lipid content was determined following the protocol established by the American Oil Chemists’ Society (AOCS) Official Procedure Am 5-04 [25] using the fat extractor Ankom XT10 (ANKOM Technology Corp., Macedon, NY, USA).

### 2.3. Amino-Acid Profile

The amino-acid profile was determined according to the protocol described by Domínguez et al. [26]. Briefly, 100 mg of samples were weighed in glass ampoules and 5 mL of 6 N hydrochloric acid solution were added. Afterward, the ampoule was sealed and kept at 110 °C for 24 h. Once the hydrolysis of proteins was completed, the hydrolysate was diluted with 200 mL of distilled water and filtered through a 0.45-µm filter (Filter Lab, Barcelona, Spain). Tryptophan was not determined due to its transformation into ammonium under acidic conditions. The derivatization of standards and samples was performed as follows: 10 µL of sample was buffered to pH 8.8 (AccQ-Fluor borate buffer) to yield a total volume of 100 µL. Then, 20 µL of AccQ-Fluor reagent (3 mg/mL in acetonitrile) was added in order to obtain a rapid derivatization of all primary and secondary amines. Excess reagent was hydrolyzed within 1 min. In order to complete the derivatization for tyrosine phenol modification, the vials were heated at 55 °C for 10 min.

The separation of derivatized amino acids was performed by high-performance liquid chromatography (Alliance 2695 model, Waters, Milford, MA, USA) using a scanning fluorescence detector (model 2475, Waters). The column used was a Waters AccQ-Tag column (3.9 × 150 mm, with a particle size of 3 µm). The chromatography conditions were as follows: flow rate of 1.0 mL/min at 37 °C. The wavelengths for excitation and emission were set at 250 and 395 nm, respectively. The mobile phase composition and the gradient were defined according to the validated and patented method of Waters Corporation (AccQ-Tag Amino acid analysis protocol). Briefly, the mobile phase was composed of three solvents: (A) AccQ Tag Eluent A concentrate for amino acids analysis (Waters, Milford, MA, USA), (B) acetonitrile (HPLC grade, Scharlab, Barcelona, Spain), and (C) ultra-pure water (Milli-Q system, Millipore, Darmstadt, Germany). The solvent gradient was set as follows: 0.0–17.0 min 99% A and 1% B; 17.0–22.0 min 96% A and 4% B; 22.0–24.0 min 95% A and 5% B; 24.0–31.5 min 91% A and 9% B; 31.5–36.0 min 83% A and 17% B; 36.0–42.0 min 83% A and 17% B; 42.0–44.0 min 60% B and 40% C; 42.0–45.0 min 99% A and 1% B.

The identification of amino acids was carried out by comparing the retention times. The quantification was performed using the external standard technique with an amino-acid standard (Amino Acid Standard H, Thermo Scientific, Rockford, IL, USA). The system operation and data acquisition were performed using the Empower 2 advanced software (Waters). The results were expressed as g/100 g of protein.

Once the amino-acid profile was determined, the sum of essential (EAA) and non-essential (NEAA) amino acids, as well as the EAA/NEAA ratio, was calculated. The EAA score for each EAA was determined according to the equation described by Domínguez et al. [26] considering the concentration of a given EAA in the sample (EAAt, g/100 g protein) and the pattern concentration (EAAp, g/100 g protein) according to World Health Organization/Food and Agriculture Organization/United Nations University (WHO/FAO/UNU) [27].

(1) EAA score = [(EAAt)/(EAAp)] × 100

The EAA index for each section of sea bass was determined according to the equation described by Domíngez et al. [26], which takes into account EAAt and EAAp (according to WHO/FAO/UNU [27]) from *i* to *n* EAA quantified in the sample.

(2) $$EAA\;index\;=\;100\;\times \;\sqrt[{n}]{\frac{EAAt_{i}}{EAAp_{i}}\;+\;\frac{EAAt_{j}}{EAAp_{j}}\;+\;\cdots \;+\;\frac{EAAt_{n}}{EAAp_{n}}}$$

### 2.4. Fatty-Acid Profile

In order to determine the fatty-acid profile of sea bass fillets and by-products, the fatty acids were obtained using the protocol described by Bligh and Dyer [28] with modification proposed by Barros [29]. Once the fatty acids were extracted, these compounds were transesterified according to the method described by Domínguez et al. [30] with some modifications. Briefly, 1 mL of toluene was used to dissolve 20 mg of fat prior to mixing with 2 mL of 0.5 N sodium methoxide solution in a test tube. This mixture was vortexed for 10 s and rested for 15 min at room temperature. Afterward, 4 mL of 10% of H_2_SO_4_ methanolic solution was added to the mixture that was vortexed for a few sec. Then, the mixture was vortexed again for a few seconds after the addition of 2 mL of saturated sodium bicarbonate solution. Subsequently, the fatty acid methyl esters (FAMEs) were separated by adding 1 mL of hexane to the tube and vortexing for 10 s. Finally, the FAMEs were transferred to an appropriate GC vial.

The FAMEs were separated and quantified by gas chromatography (GC-Agilent 7890B, Agilent Technologies, Santa Clara, CA, USA) equipped with a PAL RTC-120 autosampler and a flame ionization detector (FID). The injection was carried out in split mode (1:50) with 1 μL, the injector was kept at 260 °C, and the total flow was set to 64.2 mL/min. The separation was carried in an SP-2560 fused silica capillary column (100 m, 0.25 mm inner diameter (i.d.), 0.25-μm film thickness; Supelco Inc., Bellefonte, PA, USA). The selected carrier gas was helium with a flow rate of 1.2 mL/min. The pressure in the head of column was set to 42.135 psi. The chromatography conditions were set as follows: initial oven temperature of 140 °C (held for 4 min), first ramp at 5 °C/min to 190 °C, second ramp at 2 °C/min to 210 °C (held for 4 min), third ramp at 1 °C/min to 220 °C and fourth ramp at 3 °C/min to a final temperature of 235 °C (held for 7 min). The operational in FID was set as follows: temperature of 260 °C, flow of H_2_ 35 mL/min, air 350 mL/min, and make-up flow of 15 mL/min. The total time for chromatographic analysis was 50 min.

The software MassHunter GC/MS Acquisition B.07.05.2479 (Agilent Technologies, Santa Clara, CA, USA) was used to control the equipment and acquire the data. The data analysis was performed in the software MassHunter Quantitative Analysis B.07.01. Authenticated standards (FAME Mix, 37 components, docosapentaenoic acid, *trans*-vaccenic acid, *cis*-vaccenic acid, and CLA) were used to identify the FAMEs by comparing the retention times. The results were expressed as g/100 g of total identified fatty acids. After obtaining the fatty-acid data, the fractions of saturated (SFA), monounsaturated (MUFA), PUFA, n-3, and omega-6 (n-6) fatty-acids content were determined. The nutritional ratios PUFA/SFA and n-6/n-3 were calculated.

### 2.5. Mineral Profile

The mineral profile was determined using 5 g of ashes (obtained using the protocol ISO 936:1998 [24]) dissolved in 10 mL of 1 M HNO_3_. The assay was carried out using inductively coupled plasma optical emission spectroscopy (ICP-OES) in order to determine the concentration of Ca, K, Mg, Na, P, Fe, Mn, Zn, and Cu following the protocol defined by Lorenzo et al. [31]. The equipment used was the Thermo-Fisher ICAP 6000 plasma emission spectrometer (Thermo-Fisher, Cambridge, UK) with a radio frequency source set to 27.12 MHz, a peristaltic pump, a spraying chamber, and a concentric spray nebulizer, using 99.996% liquid argon plasma gas (Praxair, Madrid, Spain). The ICP software was used to control the system and acquire the data. The external standard procedure was used to determine the concentration of each mineral (average value of three determinations). The results were expressed as mg/100 g of meat.

### 2.6. Statistical Analysis

The statistical evaluation of data was carried out using the IBM SPSS Statistics 21 software (SPSS Inc., Armonk, NY, USA). Normal distribution and homogeneity of variance were previously tested. Significant differences were determined using either the analysis of variance (ANOVA; normal distribution of data and homogeneity of variance) or the Kruskal–Wallis test (non-normality and heterogeneity of variance). The Duncan test was applied for the data analyzed by ANOVA whereas the Dunn’s test was applied to data evaluated by Kruskal–Wallis test in the case of significant differences (*p* < 0.05). The values were presented as mean values and standard errors of the mean (SEMs).

## 3. Results

### 3.1. Chemical Composition

The evaluation of chemical composition of sea bass fillet and by-products is shown in Table 1. The evaluation of moisture content in the fillet and by-product of sea bass indicated that the higher moisture content (*p* < 0.05) was observed on the fillets. In the case of fat, gut contained more lipids (*p* < 0.05) than the fillets and other by-products. Regarding the protein content, the highest amount was obtained from the skin. Particularly for ash content, the head displayed the highest content.

### 3.2. Amino-Acid Profile

**Figure 1:**
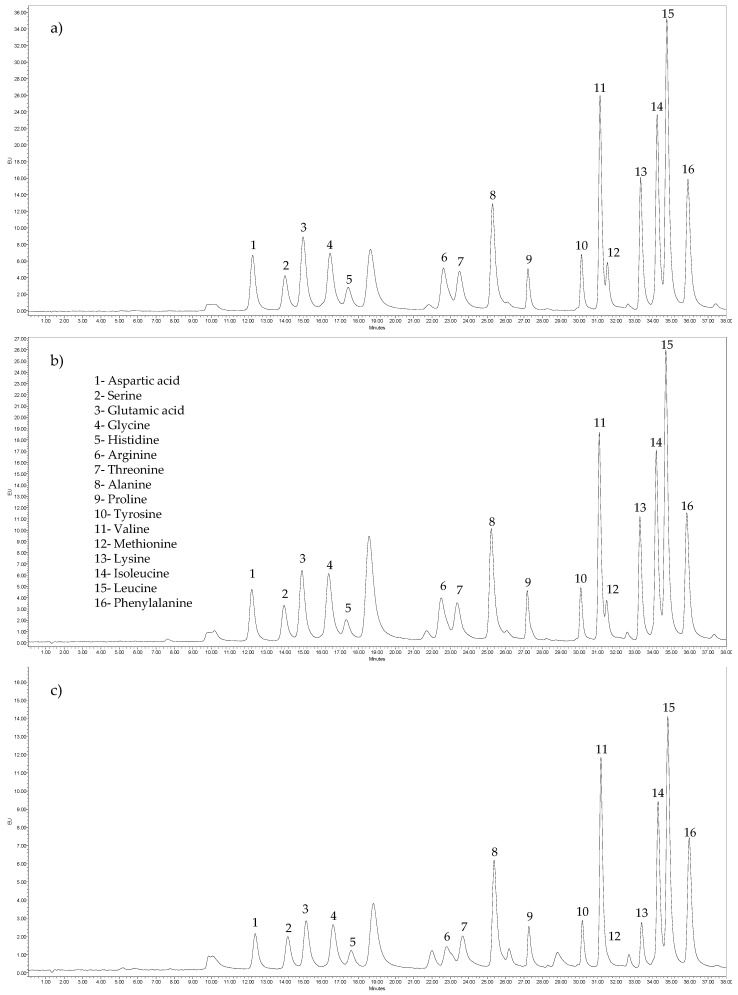
Chromatograms for the amino acid analysis: (**a**) fillet; (**b**) skin; (**c**) liver.

The evaluation of amino-acid profile indicated that fillet and skin were the main sources of both essential (8.4 and 7.9 g/100 g protein, respectively) and non-essential (8.4 and 10 g/100 g protein, respectively) amino acids (*p* < 0.05) in comparison to other samples (ranges of 0.9–6.6 and 1.3–7.8 g/100 g protein for total EAA and NEAA, respectively) (Table 2). Moreover, glutamic acid was the main amino acid among fillet, skin, guts, liver, head, and fish bone samples. It is relevant to mention that glycine was a major amino acid for skin, gills, head, and fish bone. Particularly for gills, the main amino acid was glycine, followed by glutamic acid and proline. Regarding the EAA/NEAA ratio, the highest ratio was obtained from fillet, followed by liver and fish bone. Taurine and cysteine were not determined. Figure 1 shows representative chromatograms from the amino-acid analysis.

The evaluation of amino-acid score (Table 3) indicated that the largest values for methionine, lysine, isoleucine, leucine, and valine were obtained from fillets (*p* < 0.05). In the case of histidine and threonine, the main sources were fish bone and gills, respectively (*p* < 0.05). Concerning the EAA index, the highest score was observed in the fillets, followed by skin and liver.

### 3.3. Fatty-Acid Profile

The evaluation of fatty-acid profile indicated significant differences (*p* < 0.05) among samples regarding the fractions and individual fatty acids (Table 4). The highest PUFA content (*p* < 0.05) was observed in the head and fish bones, followed by fillets and skin. In the case of MUFA fraction, all the by-products displayed higher amounts (*p* < 0.05) than fillets. Moreover, the liver was the main source of MUFA. Regarding the SFA content, the lowest amounts (*p* < 0.05) were obtained from head and fish bones, followed by guts, fillets, and skin.

**Figure 2:**
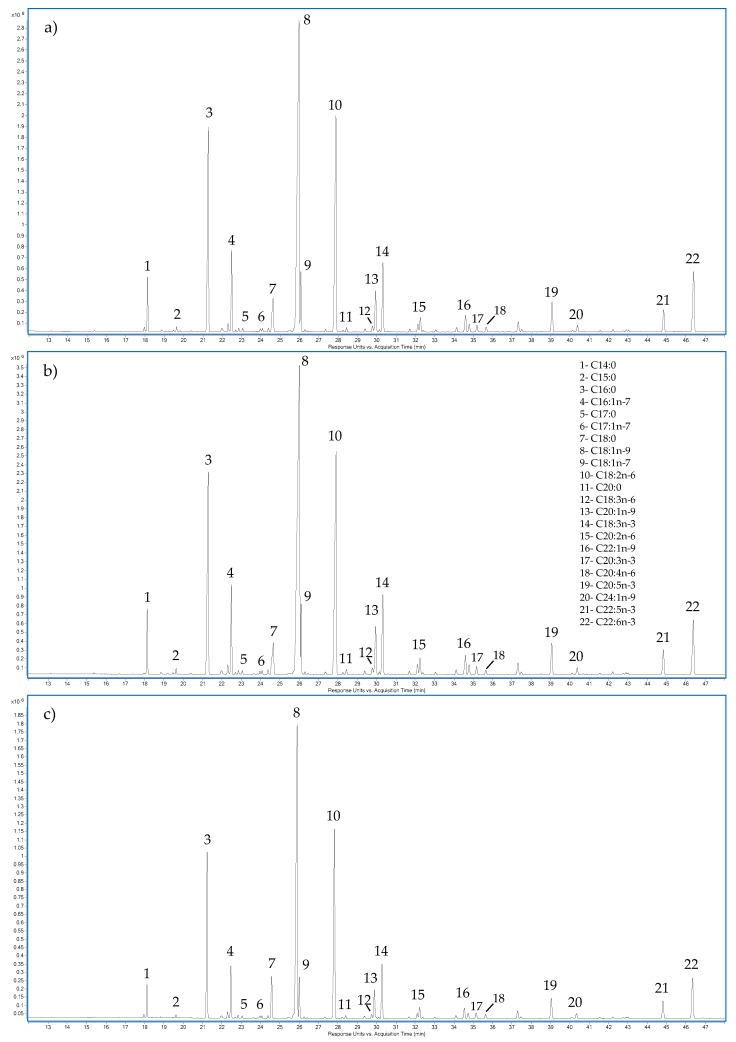
Chromatogram for fatty-acid analysis: (**a**) fillet; (**b**) skin; (**c**) liver.

Concerning the individual fatty acids, a common outcome was observed for all samples; the main fatty acids were C18:1n-9 (oleic acid), C16:0 (palmitic acid), and C18:2n-6 (linoleic acid). In addition, the long chain n-3 fatty acids eicosapentaenoic acid (C20:5n-3, EPA) and docosahexaenoic acid (C22:6n-3, DHA) were present in all samples, particularly in the fillets. In the case of n-3 content, the highest amount was obtained from fillets, followed by skin. A different scenario was observed for the n-6 content, wherein the highest concentration of this group of fatty acids was obtained from the fish bone, followed by head and guts. In addition, significant differences (*p* < 0.05) in the n-6/n-3 ratio among samples were obtained. The lowest n-6/n-3 ratio was observed on fillet, followed by skin and gill samples. Figure 2 shows representative chromatograms from the fatty-acid analysis.

### 3.4. Mineral Profile

The mineral content of sea bass fillets and by-products is displayed in the Table 5. The highest concentrations of calcium and phosphorus were obtained from head samples, followed by fish bone and gills. In the case of potassium, fillet and fish bone were the samples with highest contents, followed by liver, head, skin, and gills. Regarding magnesium, the fillets, skin, guts, and gills had the highest concentrations. Particularly for sodium, the gills had the highest content. In the case of microminerals, the liver was the sample with the highest amount of copper, iron, and zinc, whereas the highest content of manganese was observed in the gills.

## 4. Discussion

### 4.1. Chemical Composition

Scientific information about the chemical composition, fatty acid, protein, and mineral profile of fish by-products is scarce, reported for few species and using a different approach to obtain the fillets and dissect internal organs. Consequently, this scenario complicates the comparison across studies. Regarding the composition of sea bass fillets observed in the present study, these values were the ranges of values reported in a study about the effect of seasonal changes on sea bass fillets [32]. The animals fished during fall and winter displayed higher moisture values than samples obtained using spring and summer. Conversely, an opposite trend was observed for protein content between these two groups of seasons. It is also relevant to mention that the range for values obtained for moisture, protein, and fat (in the ranges of 62.9%–78.2%, 17.1%–20.8%, and 0.9%–16.4%, respectively) were in the range of values reported for sea bass reared in different systems (extensive, semi-extensive, intensive, and sea-cages) [33].

Concerning the composition of sea bass by-products, a similar pattern for the chemical components of dissected fish can be observed; viscera and/or carcass contain relevant amounts of protein and fats. This outcome is supported by the outcomes obtained with rainbow trout (*Oncorhynchus mykiss*) [34], blunt snout bream (*Megalobrama amblycephala*) [35], and olive flounder (*Paralichthys olivaceus*) [36] in comparison to the respective fillet or whole-fish composition. It is also relevant to mention that, to the best of our knowledge, the sea bass by-products evaluated in this study were collectively discarded without any separation into organs at industrial level.

### 4.2. Protein and Amino-Acid Profile

The amino-acid profile of sea bass indicated that the predominant amino acids were glutamic acid and glycine for most of the samples. Although the information regarding the amino-acid profile of sea bass is scarce and used the whole fish as a sample, the results obtained in the present study are in accordance with the experiment carried out by Özyurt and Polat [32]. These authors observed that glutamic acid was the predominant amino acid (from 7.69 to 9.61 g/100 g protein) in the composition of sea bass throughout the year. However, aspartic acid and lysine (in ranges of 8.25–9.14 and 7.43–9.49 g/100 g protein) were also indicated as main amino acids. Regarding the EAA/NEAA ratio, this index ranged from 0.75 to 0.77. The predominance of glutamic acid in sea bass was reported in another study that aimed to compare the differences between wild and farmed rearing systems (1475.00 vs. 2369.00 mg/100 g for wild and farmed sea bass, respectively) [37]. Interestingly, this study indicated lysine, aspartic acid, and leucine as the main amino acids in both farmed and wild sea bass. In relation to the EAA/NEAA ratio, the authors obtained values of 1.10 and 1.20 for farmed and wild fishes, respectively.

The analysis of EAA score is an important indicator to evaluate in depth the content of each one of these compounds. It is important to mention that only sea bass fillet displayed adequate values for all the amino acids (scores above 100; Table 3). This outcome is in agreement with the data obtained for *Clupea harengus*, *Scomber scombrus*, *Trachurus trachurus*, and *Urophycis tenuis* fresh fillets by achieving adequate scores for all EAA [38]. A similar outcome was reported for yellowfin (*Thunnus albacares*) and bigeye tuna (*Thunnus obesus*) fresh fillets in relation to the EAA scores [39]. However, the limiting EAA varies among fish species. These contrasting results were reported for the fillets *Clarias anguillaris*, *Oreochromis niloticus*, and *Cynoglossus senegalensis* [40]. In these three species, the limiting amino acid was threonine. Moreover, lower scores than those recommended by FAO/WHO were reported for isoleucine, leucine, lysine, methionine + cysteine, and valine by the authors.

In the case of sea bass by-products, methionine was the only limiting amino acid. Although the results obtained in the present study support the quality of EAA of sea bass by-products, the information regarding this aspect of fish by-products is limited and reported for other species. The study carried out with silver carp (*Hypophthalmichthys molitrix*) indicated histidine as the limiting EAA for the by-product portion (composed of bones, cutoffs, dark muscle, fins, frame, scales, skin, and viscera) [41].

Regarding the EAA index, previous studies did not characterize this parameter for sea bass fillet and by-products. However, some studies reported the EAA index for other fish species. The EAA index was determined for *Lates niloticus* and *Oreochromis niloticus* fillets and whole-body composition of *Rastrineobola argentea*, *Limnothrissa miodon*, and *Stolothrissa tanganicae* [42]. According to the authors, the index varied between 92 (*Limnothrissa miodon*) and 102 (*Oreochromis niloticus*). In the experiment carried out by Wu and Mao [43], the EAA index of grass carp (*Ctenopharyngodon idellus*) fillets was 0.87 (or 87 using the equation of the present study). Thus, this scenario strengthens the biological importance of sea bass by-products as a source of EAA.

The characterization of the sea bass by-products also revealed an interesting scenario for their further use to elaborate functional ingredients and foods to prevent nutritional deficiencies and improve the supply of products with health claims. The nutritional and health claims from the European regulation [44] provide a relevant approach to evaluate the composition of sea bass by-products and indicate potential uses in the development of functional additives.

In relation to the protein content, the skin, gills, head, and fish bone of sea bass can be considered as the most promising “source of protein” or being labeled as “high in protein” among the by-products evaluated in this study (energy associated with protein contain superior to 12% and 20%, respectively). In this scenario, these fractions of sea bass by-products could be considered “high in protein”. It is also relevant to mention that the liver could be considered as a “source of protein”. Conversely, the guts did not achieve the limit to be considered a “source of protein”. In this sense, a potential application of this by-product could be the fortification of baked products [11].

### 4.3. Fat and Fatty Acids

The analysis of fatty-acid profile of sea bass fillets and by-products revealed a relevant proportion of MUFA and PUFA. Although other studies support the high proportion of unsaturated fatty acids (UFA) in the composition of sea bass [45,46,47], the proportion of each fraction varies across studies. This scenario can be observed in the study carried out by Mourente and Bell [47] who observed that MUFA was the main fraction of fatty acids, followed by PUFA in the muscles and liver of sea bass feed, with partial replacement of vegetables by fish oil. These two fractions of fatty acids accounted for more than 70% of the total fatty acids. In another experiment carried out to investigate the effect rearing system, Bhouri et al. [45] observed that PUFA was the main fraction of fatty acids, followed by MUFA in the dorsal and ventral muscles and liver of sea bass. Conversely, an experiment on the influence of iced storage on the fatty-acid profile of sea bass skin indicated that SFA was the main fraction, followed by MUFA and PUFA [46].

Regarding the individual fatty acids, the results obtained in this study are in agreement with other studies for fillets and by-products regarding the high content of oleic acid and palmitic acid (among MUFA and SFA, respectively) in the fillets, skin, and liver of sea bass [45,46,47]. However, some discrepancies can be noted regarding the main fatty acid among the PUFAs. In the present study, linoleic acid was the main fatty acid in this fraction, followed by DHA (Table 4). These results differ from those reported by other authors who indicated that DHA was the main fatty acid in the PUFA fraction in different portions of the sea bass [45,46,47,48]. A possible explanation could be related to the season of capture, since a previous study indicated that the proportion of both linoleic acid and DHA in the sea bass was influenced by the season [32]. According to the authors of that study, the concentration of DHA surpasses the concentration of linoleic acid during winter, while, during the other seasons, an opposite trend was observed.

Regarding the claims for fat composition, sea bass by-products contained relevant amounts of UFA, MUFA, and long chain n-3 fatty acids. These indicators are also present in European regulation [44] regarding nutritional and health claims. Regarding the UFA, this regulation determines the minimum limit of UFA (sum of MUFA and PUFA) as at least 70%, and more than 20% of energy must be derived from this combination of fractions for the use of a “high unsaturated fat” claim. In this sense, all by-products overcame both limits and could be considered as “high unsaturated fat” sources.

In the case of MUFA content, this regulation establishes that a “high monounsaturated fat” claim is attributed to products with MUFA proportion higher than 45% and with more than 20% of total energy provided by this fat fraction. With this line of thought, the sea bass by-products that overcame both limits for MUFA and percentual energy related to this fat fraction are guts, gills, liver, head, and fish bone, and they could be considered “high monounsaturated fat” sources. In the case of skin, the MUFA proportion did not surpass the minimum limit of 45% of MUFA in total fatty-acid content.

The same approach could also be applied for n-3 fatty acids, wherein the claim “source of n-3 fatty acids” is attributed to samples with at least 40 mg of EPA + DHA/100 g of product and per 100 kcal, and the “high n-3 fatty acid” content claim is attributed to samples with at least 80 mg of EPA + DHA/100 g of product and per 100 kcal [44]. In accordance with these limits, all by-products complied with the “high n-3 fatty acids” content claim. Following this line of thought, the incorporation of n-3 fatty acids in meat products can be seen as a promising strategy to develop healthier products [9].

### 4.4. Minerals

Only a few studies evaluated the mineral composition of sea bass wherein either fillet or whole fish was considered as a sample. Particularly for macro-minerals, the magnesium content obtained from the fillets in the present study is in agreement with the data reported by Zotos and Vouzanidou [49] for the edible portion of (fillet with skin) of sea bass. However, some differences in the content of macro-minerals can be observed in relation to studies that considered the whole fish as a sample. In the study carried out by Erkan and Özden [50], the main macro-minerals were potassium and phosphorus, followed by sodium and calcium (4597, 3736, 773, and 636 mg/kg, respectively). Particularly for by-products, a study carried out to characterize the composition of sea bass indicated that the liver of this fish was mainly composed of sodium and potassium, followed by calcium and magnesium (9580, 6350, 800, and 880 mg/kg, respectively) [45]. Significant differences between farmed and wild fishes were observed for all macro-minerals according to authors. Unlike that observed in the present study, the liver displayed a similar macro-mineral profile in comparison to fillet.

In relation to the micro-minerals, Yildiz [51] indicated that iron and zinc (in the ranges of 3.1–4.3 and 3.1–5.1 mg/kg, respectively) were among the main micro-minerals in the fillets of both farmed and wild sea bass, followed by manganese and copper (from 1.2 to 1.9 and from 0.1 to 0.3 mg/kg, respectively). Moreover, the author also observed that these minerals were predominant in fillets of sea bass regardless of changes in the micro-mineral of farmed and wild fish. Another similar outcome was reported by Zotos and Vouzanidou [49] who evaluated the effect of season on the mineral composition of the edible portion of sea bass. This study indicated that iron and zinc (in the ranges of 25–29 and 21–24 mg/kg, respectively) were the main micro-mineral components, similar to that observed in the present study. However, the authors did not observe significant differences across seasons. Unlike that observed in the present study, another study on the micro-mineral profile of sea bass liver indicated that iron and zinc were indicated as main components in this fraction of minerals (in the ranges of 134–581 and 97–132 mg/kg, respectively) [45].

It is relevant to mention that a similar outcome in relation to main micro-minerals can be observed in relation to authors that used the whole fish as a sample. A study that evaluated the differences between farmed and wild sea bass indicated iron and zinc as main micro-minerals, followed by manganese [52]. Conversely, only iron was indicated as main micro-mineral in the sea bass evaluated by Erkan and Özden [50], whereas zinc was present at low concentration (24.7 and 2.8 mg/kg, respectively).

In the case of claims for minerals, the classification of “source of” and “high content of” varies among minerals in accordance with daily recommended levels [44]; for calcium and phosphorus, the levels are 800 mg/100 g. In the case of sea bass head and fish bone, both can be considered as “sources of phosphorus” (content superior to 800 mg/100 g) and “high in calcium” (concentration higher than 1600 mg/100 g). Additionally, the gills can also be considered as a “source of Ca”. It would be interesting to explore the use of these sources of minerals in dairy products such as the use of calcium in yogurt [10].

The results obtained in this study can be used to support further experiments targeting both food applications (as indicated previously for amino acids, fatty acids, and minerals) and the development of nutraceuticals from each by-product of sea bass. A relevant example of application is the enrichment of meat products with n-3 fatty acids from sea bass liver and guts. This particular strategy combines the scientific evidence of n-3 fatty acids to promote health with the low natural n-3 fatty-acid content of meat products [9]. Alternatively, the consumption of n-3 fatty-acid capsules is an additional commercial application with health-related effects [12]. Another relevant approach is fortifying dairy products with calcium obtained from sea bass head, fish bones, and gills. In this case, calcium is a relevant component naturally found on yogurt, but increasing its content is of great value for consumers seeking to improve the daily ingestion of this mineral [10]. Likewise, these strategies can be applied for the other sea bass components evaluated in this study to develop healthier and functional food products or supplements. Furthermore, it is also relevant to mention that metals occur naturally in an aquatic system, but they can be considered as pollutants and they accumulate in the sea bass liver and other organs as a consequence of human activity (urban effluents and industrial activity, for instance). Other relevant pollutants (such as organochlorinated compounds) can also be detected in sea bass organs but at lower levels than those established by health organizations [53]. 

The economic viability of exploring sea bass by-products to obtain functional ingredients and produce nutraceuticals depends on the final products. In the case of fatty acids, the existing industrial structure, machinery, and market [6] are favorable aspects for the insertion of sea bass by-products rich in omega-3 fatty acids into the current production chain of fish oil. In the context of the present study, it seems reasonable to consider that extracting omega-3 fatty acids from sea bass by-products would be possible in the current fish industry.

A different scenario is observed for the exploration of amino acids and minerals from sea bass by-products. Particularly for amino-acid extraction from sea bass by-products, it is necessary to break down fish proteins. Using microbial enzymes can be seen as a feasible approach from an economic point of view [54]. In the case of minerals, the preparation of mineral-rich products could be obtained by simple and low-cost operations such as treating the fish bones with weak solutions (alkali, acid, and hydrogen peroxide), followed by drying and grinding [55].

Further studies are necessary to strengthen these approaches. It is also important to highlight that the reuse of sea bass by-products (discarded material rich in biologically relevant compounds) is one of the necessary steps toward a more sustainable food industry [13].

## 5. Conclusions

The evaluation of sea bass fillet and by-products indicated a wide diversity of compounds associated with human health. Taking into account the amounts and the biological significance of fatty-acid, amino-acid, and mineral profiles analyzed in this study, it is possible to suggest the use of guts and liver to obtain MUFA, UFA, and long-chain n-3 fatty acids, as well as head, fish bones, and gills to recover minerals, particularly calcium, phosphorus, and manganese, and skin to obtain proteins and amino acids.

## Figures and Tables

**Table 1:** Chemical composition of sea bass fillet and by-products.

(g/100 g)	Fillet	Skin	Guts	Gills	Liver	Head	Fish Bone
Moisture	72^e^	54^bc^	38^a^	62^de^	48^ab^	59^cd^	52^b^
SEM	0.4	1	2	0.4	1	0.8	1
Protein	21^ef^	25^f^	8^a^	16^de^	12^ab^	16^cd^	15^bc^
SEM	0.08	0.7	0.6	0.1	0.5	0.2	0.4
Fat	4^a^	17^bc^	53^e^	13^ab^	35^de^	14^ab^	19^cd^
SEM	0.5	1	3	0.5	2	0.6	0.6
Ash	1.3^bc^	3.0^cd^	0.78^a^	5.8^de^	1.1^ab^	10^f^	7^ef^
SEM	0.02	0.3	0.03	0.2	0.07	0.6	0.4

SEM: standard error of the mean; n.d.: not determined; *n* = 10. ^a–e^ Means in the same row with different letters differ significantly (*p* < 0.05; Dunn’s or Duncan’s test).

**Table 2:** Amino-acid profile of sea bass fillet and by-products.

Amino Acid (mg/100 g Protein)	Fillet	Skin	Guts	Gills	Liver	Head	Fish Bone
*EAA*							
Histidine	464^c^	534^c^	n.d.^a^	435^c^	236	455^c^	463^c^
SEM	29	40	-	16	50	17	11
Arginine	1173^bc^	1595^c^	96.41^a^	215^a^	205^a^	1189^bc^	1195^bc^
SEM	76	119	6	16	21	39	34
Threonine	798^c^	850^c^	107^a^	808^c^	286^a^	637^ab^	688^bc^
SEM	46	71	5	23	80	22	26
Valine	922^c^	897^bc^	155^a^	672^a^	510^a^	666^a^	701^ab^
SEM	49	80	4	57	97	27	25
Methionine	437^d^	247^c^	n.d.^a^	n.d.^a^	109^b^	199^bc^	197^bc^
SEM	21	33	-	-	10	9	8
Lysine	1646^d^	1532^cd^	108^a^	799^ab^	407^a^	1055^ab^	1232^bc^
SEM	99	168	4	48	147	54	58
Isoleucine	837^c^	739^bc^	112^a^	451^a^	365^a^	547^ab^	583^ab^
SEM	42	75	4	39	74	25	24
Leucine	1350^c^	1230^bc^	169^a^	7839^ab^	609^a^	899^ab^	938^ab^
SEM	68	118	15	61	128	41	39
Phenylalanine	799^c^	771.87^b^	124^a^	554^ab^	399^a^	608^ab^	601^ab^
SEM	43	60	7	24	82	22	18
Σ EAA	8427^d^	8397^cd^	872^a^	4718^ab^	3126^a^	6256^ab^	6598^bc^
SEM	465	723	126	293	656	231	222
*NEAA*							
Aspartic acid	1638^cd^	1695^d^	225^a^	1001^ab^	520^ab^	1258^abc^	1350^bcd^
SEM	91	160	1	63	68	53	50
Serine	686^b^	841^b^	129^a^	624^ab^	361^ab^	692^b^	679^b^
SEM	45	64	11	29	74	23	19
Glutamic acid	2486^c^	2546^d^	340^a^	1559^ab^	913^ab^	1887^b^	1985^bc^
SEM	142	236	25	94	89	79	72
Glycine	975^b^	2086^c^	252^a^	1706^c^	434^ab^	1651^c^	1469^c^
SEM	68	225	56	12	141	87	71
Alanine	864^ab^	1336^c^	198^a^	1048^bc^	510^a^	927^bc^	954^bc^
SEM	42	139	0.8	54	131	40	33
Proline	551^bc^	1208^e^	141^a^	1097^de^	402^ab^	957^de^	818^cd^
SEM	32	145	27	52	56	60	35
Tyrosine	660^c^	524^bc^	40^a^	299^a^	203^a^	413^ab^	416^ab^
SEM	40	50	0.5	16	36	20	18
Σ NEAA	7860^b^	10237^c^	1324^a^	7334^ab^	3344^a^	7784^b^	7671^b^
SEM	451	844	160	315	385	271	207
EAA/NEAA	1.07^d^	0.82^abc^	0.66^ab^	0.641^a^	0.93^cd^	0.80^ab^	0.86^bc^
SEM	0.006	0.03	0.01	0.004	0.05	0.02	0.02

SEM: standard error of the mean; EAA: essential amino acid; NEAA: non-essential amino acid; n.d.: not determined; *n* = 10. ^a–e^ Means in the same row with different letters differ significantly (*p* < 0.05; Dunn’s or Duncan’s test).

**Table 3:** Amino-acid scores and index of sea bass fillet and by-products.

EAA	Fillet	Skin	Guts	Gills	Liver	Head	Fish Bone
Histidine	148^bc^	147^b^	n.d.^a^	174^bcd^	154^bc^	198^cd^	217^d^
SEM	9	12	-	19	5	8	6
Threonine	255^de^	235^cde^	81^a^	323^e^	186^abc^	181^ab^	211^bcd^
SEM	14	23	2	36	4	7	8
Valine	295^b^	249^b^	118^a^	269^b^	331^b^	111^a^	127^a^
SEM	15	26	6	40	15	5	5
Methionine	140^c^	69.15^b^	n.d.^a^	n.d.^a^	71^b^	81^b^	87^b^
SEM	6	11	-	-	4	4	4
Lysine	526^c^	426^c^	82^a^	322^bc^	265^bc^	153^a^	193^ab^
SEM	31	54	5	34	17	9	9
Isoleucine	267^b^	205^b^	85^a^	180^ab^	238^b^	119^a^	137^a^
SEM	13	24	5	30	12	6	6
Leucine	431^d^	341^bc^	129^abc^	314^bcd^	397^cd^	99.43^a^	112^ab^
SEM	21	39	15	31	21	5	5
EAA index	266^c^	208^bc^	26^a^	115^a^	208^bc^	128^a^	147^ab^
SEM	14	23	1	9	14	6	5

SEM: Standard error of the mean; n.d.: not determined; *n* = 10. ^a–e^ Means in the same row with different letters differ significantly (*p* < 0.05; Dunn’s or Duncan’s test). Limiting amino acids are indicated in bold letters.

**Table 4:** Fatty-acid profile of sea bass fillet and by-products.

Fatty Acid (g/100 g Fatty Acids)	Fillet	Skin	Guts	Gills	Liver	Head	Fish Bone
C14:0	3.2^bc^	3.4^cd^	3.9^e^	3.5^de^	2.2^a^	2.4^a^	2.47^ab^
SEM	0.03	0.03	0.05	0.05	0.07	0.01	0.006
C15:0	0.298^b^	0.307^b^	0.320^bc^	0.342^c^	0.224^a^	0.257^a^	0.250^a^
SEM	0.003	0.004	0.007	0.006	0.01	0.001	0.0006
C16:0	17^cd^	17^cd^	17^bc^	19^de^	20^e^	16^ab^	15^a^
SEM	0.1	0.2	0.2	0.1	0.3	0.04	0.03
C16:1n-7	4.3^bc^	4.6^cd^	5.2^de^	5.3^e^	4.0^ab^	4.0^ab^	3.96^a^
SEM	0.06	0.08	0.05	0.05	0.10	0.01	0.004
C17:0	0.24^a^	0.253^ab^	0.240^ab^	0.257^b^	0.318^c^	0.495^d^	0.501^d^
SEM	0.01	0.003	0.003	0.003	0.008	0.002	0.003
C17:1n-7	0.192^b^	0.204^b^	0.211^bc^	0.253^cd^	0.248^cd^	0.00^a^	0.366^d^
SEM	0.007	0.007	0.003	0.008	0.008	0.00	0.005
C18:0	3.63^d^	3.40^cd^	3.14^bc^	2.93^ab^	5.23^d^	2.82^a^	3.09^bc^
SEM	0.07	0.07	0.08	0.07	0.45	0.008	0.01
C18:1n-9	29^a^	31^ab^	32^b^	32^b^	38^d^	34^c^	34^c^
SEM	0.3	0.2	0.2	0.2	0.7	0.04	0.03
C18:1n-7	2.8^a^	3.0^bc^	3.2^c^	3.1^c^	3.3^d^	2.83^ab^	2.7^a^
SEM	0.04	0.01	0.03	0.03	0.2	0.003	0.02
C18:2n-6	11^ab^	12^bc^	13^cd^	11^ab^	9^a^	17^de^	18^e^
SEM	0.09	0.1	0.1	0.1	0.4	0.02	0.02
C20:0	0.233^bc^	0.243^cd^	0.265^d^	0.245^cd^	0.186^a^	0.199^a^	0.211^ab^
SEM	0.003	0.004	0.005	0.004	0.005	0.001	0.002
C18:3n-6	0.210^a^	0.223^b^	0.224^b^	0.208^a^	0.272^c^	0.289^d^	0.265^c^
SEM	0.002	0.002	0.002	0.002	0.009	0.003	0.002
C20:1n-9	4.0^bc^	4.3^cd^	5^d^	4.2^bc^	3.0^ab^	2.40^a^	2.48^a^
SEM	0.05	0.05	0.1	0.04	0.04	0.004	0.004
C18:3n-3	3.0^bc^	3.1^c^	3.3^cd^	2.7^ab^	2^a^	3.69^de^	3.77^e^
SEM	0.03	0.03	0.05	0.03	0.1	0.007	0.006
C20:2n-6	0.67^abc^	0.70^bc^	0.73^c^	0.63^ab^	0.55^a^	1.069^d^	1.060^d^
SEM	0.01	0.01	0.02	0.01	0.01	0.004	0.003
C22:1n-9	0.418^bc^	0.450^cd^	0.51^de^	0.420^bc^	0.29^ab^	0.257^a^	0.668^e^
SEM	0.006	0.006	0.02	0.007	0.01	0.002	0.002
C20:3n-3	0.213^bc^	0.221^c^	0.232^c^	0.178^ab^	0.148^a^	0.289^d^	0.277^d^
SEM	0.007	0.004	0.005	0.002	0.006	0.001	0.002
C20:4n-6	0.58^c^	0.446^ab^	0.359^a^	0.469^b^	0.31^a^	0.548^c^	0.484^b^
SEM	0.02	0.006	0.003	0.009	0.02	0.001	0.001
C20:5n-3	5.6^e^	5.1^de^	4.2^bc^	4.4^cd^	3^a^	3.5^ab^	3.37^a^
SEM	0.07	0.06	0.08	0.07	0.2	0.01	0.005
C24:1n-9	0.361^bc^	0.38^cd^	0.41^d^	0.379^cd^	0.27^ab^	0.232^a^	0.212^a^
SEM	0.006	0.06	0.01	0.008	0.01	0.002	0.003
C22:5n-3	1.2^c^	1.2^c^	1.1^b^	0.96^ab^	0.84^a^	1.20^c^	1.06^ab^
SEM	0.02	0.02	0.02	0.02	0.05	0.007	0.004
C22:6n-3	9^e^	7^de^	5.3^ab^	6.5^cd^	4.5^ab^	5.5^bc^	4.4^a^
SEM	0.2	0.1	0.09	0.15	0.3	0.03	0.02
SFA	25^bc^	25^bc^	24^b^	26^cd^	29^d^	22^a^	22^a^
SEM	0.1	0.2	0.3	0.2	0.65	0.06	0.04
MUFA	41^a^	43^b^	46^d^	46^cd^	49^d^	43^ab^	44^bc^
SEM	0.3	0.2	0.1	0.2	0.5	0.04	0.04
PUFA	32^cd^	30^bc^	28^ab^	27^a^	22^a^	34^e^	33^de^
SEM	0.3	0.3	0.3	0.3	1	0.06	0.03
n-3	19^d^	17^cd^	14^b^	15^bc^	11^a^	13^a^	13^a^
SEM	0.3	0.2	0.2	0.3	0.7	0.05	0.03
n-6	13^bc^	13^bc^	14^cd^	12^ab^	11^a^	19^de^	20^e^
SEM	0.09	0.1	0.2	0.1	0.4	0.02	0.02
n-6/n-3	0.67^a^	0.76^ab^	1.0^cd^	0.84^bc^	0.99^cd^	1.37^de^	1.55^e^
SEM	0.01	0.01	0.01	0.01	0.03	0.004	0.004
Long chain n-3	15^e^	14^de^	11^bc^	12^cd^	8^a^	10^ab^	8.9^a^
SEM	0.3	0.2	0.1	0.2	0.5	0.04	0.03

SEM: Standard error of the mean; SFA: saturated fatty acid; MUFA: monounsaturated fatty acid; PUFA: polyunsaturated fatty acid; n.d.: not determined; *n* = 10. ^a–e^ Means in the same row with different letters differ significantly (*p* < 0.05; Dunn’s or Duncan’s test).

**Table 5:** Mineral profile of sea bass fillet and by-products.

Mineral	Fillet	Skin	Guts	Gills	Liver	Head	Fish Bone
*Macro-minerals (mg/100 g)*							
Calcium	32^ab^	735^bc^	26^ab^	1382^cd^	9^a^	2507^e^	2093^de^
SEM	4	108	3	47	1	116	121
Magnesium	34^c^	37^c^	34^c^	37^c^	20^a^	29^bc^	25^ab^
SEM	0.9	4	3	0.9	2	2	0.6
Phosphorus	206^bc^	468^cd^	113^a^	743^de^	175^b^	1277^e^	1166^e^
SEM	3	53	4	23	8	58	60
Potassium	306^c^	189^b^	87^a^	180^b^	242^b^	194^b^	263^c^
SEM	4	7	4	5	45	3	10
Sodium	139^b^	161^b^	144^b^	251^c^	163^b^	163^b^	96^a^
SEM	4	8	7	5	13	6	3
*Micro-minerals (mg/100 g)*							
Copper	0.112^b^	0.15^c^	0.42^d^	0.094^ab^	14^e^	0.034^a^	0.110^b^
SEM	0.003	0.01	0.04	0.008	2	0.001	0.008
Iron	0.55^b^	0.53^b^	1.03^c^	1.23^c^	2^d^	0.29^a^	0.52^ab^
SEM	0.05	0.03	0.07	0.06	0.2	0.04	0.03
Zinc	0.47^a^	2^cd^	1.2^ab^	1.4^bc^	4.13^d^	2.1^cd^	1.3^b^
SEM	0.01	0.3	0.06	0.03	0.3	0.06	0.04
Manganese (μg/100 g)	20^a^	184^bc^	145^b^	500^d^	110^ab^	267^cd^	270^cd^
SEM	1	36	17	52	9	18	7

SEM: Standard error of the mean; n.d.: not determined; *n* = 10. ^a–e^ Means in the same row with different letters differ significantly (*p* < 0.05; Dun or Duncan test).
